# Omentin protects against LPS-induced ARDS through suppressing pulmonary inflammation and promoting endothelial barrier via an Akt/eNOS-dependent mechanism

**DOI:** 10.1038/cddis.2016.265

**Published:** 2016-09-08

**Authors:** Di Qi, Xumao Tang, Jing He, Daoxin Wang, Yan Zhao, Wang Deng, Xinyu Deng, Guoqi Zhou, Jing Xia, Xi Zhong, Shenglan Pu

**Affiliations:** 1 Department of Respiratory Medicine, Second Affiliated Hospital of Chongqing Medical University, Chongqing, China

## Abstract

Acute respiratory distress syndrome (ARDS) is characterized by increased pulmonary inflammation and endothelial barrier permeability. Omentin has been shown to benefit obesity-related systemic vascular diseases; however, its effects on ARDS are unknown. In the present study, the level of circulating omentin in patients with ARDS was assessed to appraise its clinical significance in ARDS. Mice were subjected to systemic administration of adenoviral vector expressing omentin (Ad-omentin) and one-shot treatment of recombinant human omentin (rh-omentin) to examine omentin's effects on lipopolysaccharide (LPS)-induced ARDS. Pulmonary endothelial cells (ECs) were treated with rh-omentin to further investigate its underlying mechanism. We found that a decreased level of circulating omentin negatively correlated with white blood cells and procalcitonin in patients with ARDS. Ad-omentin protected against LPS-induced ARDS by alleviating the pulmonary inflammatory response and endothelial barrier injury in mice, accompanied by Akt/eNOS pathway activation. Treatment of pulmonary ECs with rh-omentin attenuated inflammatory response and restored adherens junctions (AJs), and cytoskeleton organization promoted endothelial barrier after LPS insult. Moreover, the omentin-mediated enhancement of EC survival and differentiation was blocked by the Akt/eNOS pathway inactivation. Therapeutic rh-omentin treatment also effectively protected against LPS-induced ARDS via the Akt/eNOS pathway. Collectively, these data indicated that omentin protects against LPS-induced ARDS by suppressing inflammation and promoting the pulmonary endothelial barrier, at least partially, through an Akt/eNOS-dependent mechanism. Therapeutic strategies aiming to restore omentin levels may be valuable for the prevention or treatment of ARDS.

Acute respiratory distress syndrome (ARDS) is a devastating condition with a 30–60% mortality rate.^1, 2^ Although the pathogenesis of ARDS is complex, the inflammatory response and endothelial barrier disruption play important roles in the development of ARDS.^3, 4, 5^ Therefore, in addition to conventional anti-inflammatory treatments, therapeutic strategies aim to restore pulmonary endothelial barrier integrity and function through regulating inter-endothelial AJs and the endothelial cytoskeleton to minimize protein leakage and leukocyte infiltration under ARDS conditions.^6, 7^

Obesity, especially visceral obesity, has clearly been shown to impair systemic vasculature and to lead to the initiation and progression of vascular disorders.^8, 9, 10^ Although different from the well-documented impacts of obesity on cardiovascular disease, the relationships between obesity and ARDS have not been well elucidated. Clinical and experimental data focused on pertinent physiological changes in obesity indicate that the obesity may alter ARDS pathogenesis by ‘priming' the pulmonary endothelial barrier for insult and amplifying the early inflammatory response, thus lowering the threshold to initiate ARDS.^11, 12^ Contrary to conventional dogma, adipose tissue is now appreciated as an important endocrine tissue that secretes various bioactive molecules called adipokines, which contribute to the progression of diverse vascular diseases, including hypertension, cardiovascular disease and atherosclerosis.^13, 14, 15, 16^ Although ARDS is not a classified pulmonary vascular disease, it is a severe inflammatory lung condition with widespread pulmonary endothelial breakdown. Clinical evidence has indicated that the obesity might be an emerging risk factor for ARDS and that circulating adipokines levels are associated with the initiation and progression of ARDS.^11, 12, 17, 18^ Moreover, experimental studies have suggested that some anti-inflammatory adipokines, such as adiponectin and apelin, exert beneficial actions on ARDS.^19, 20, 21^

Omentin is an anti-inflammatory adipokine that is abundant in human visceral fat tissue.^22, 23^ Paradoxically, higher circulating omentin-1 levels are present in lean and healthy individuals compared with the obese and diabetic patients. Moreover, as a novel biomarker of endothelial dysfunction, reduced circulating omentin levels are related to the pathological mechanism of obesity-linked vascular disorders, including type 2 diabetes, atherosclerosis, hypertension and cardiovascular disease.^24, 25, 26, 27, 28^ Furthermore, experimental studies have found that omentin stimulates vasodilation in isolated blood vessels and suppresses cytokine-stimulated inflammation in endothelial cells (ECs).^29, 30, 31^ Thus, these data suggest that omentin may protect against obesity-related vascular complications through its anti-inflammatory and vascular-protective properties; however, little is known regarding its role in lung tissue. It was reported that decreased circulating omentin-1 levels could be regarded as an independent predictive marker for the obstructive sleep apnea syndrome and that omentin protects against pulmonary arterial hypertension through inhibiting vascular structure remodeling and abnormal contractile reactivity.^32, 33, 34^ However, to our knowledge, no study has assessed the impact of omentin on ARDS.

Akt-related signaling pathways function as an endogenous negative feedback mechanism in response to the injurious stimulus. Our prior studies have demonstrated that Akt-related signaling contributes to protection against ARDS.^35, 36^ Moreover, omentin has been reported to exert anti-inflammatory, pro-survival and pro-angiogenic functions in various cells *via* an Akt-dependent mechanism.^30, 31, 37, 38, 39, 40, 41, 42^

Collectively, given that ARDS is ultimately an obesity-related disorder of vascular function and that omentin is a favorable pleiotropic adipokine capable of anti-inflammatory, pro-angiogenic and anti-apoptotic abilities; omentin may exert beneficial effects on ARDS. In the present study, we first aimed to appraise the clinical significance of omentin in ARDS and then specifically evaluated its impact on inflammation and the endothelial barrier. Furthermore, we mechanistically investigated the role of Akt-related signaling pathways in these effects induced by omentin *in vivo* and *in vitro*.

## Results

### Circulating omentin levels are associated with inflammation in patients with ARDS

**Figure 1:**
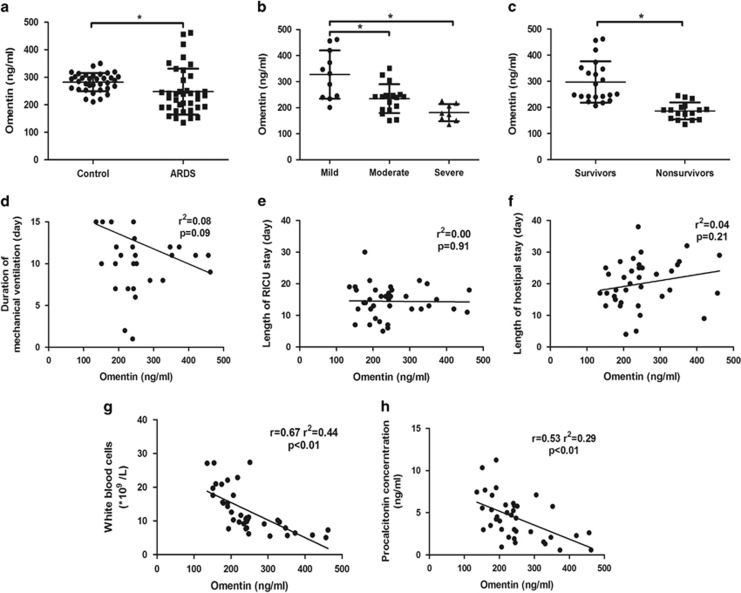
Circulating omentin levels are associated with the inflammation in patients with acute respiratory distress syndrome (ARDS). (**a**) Plasma omentin concentrations were lower in patients with ARDS (*n*=38) than in healthy controls (*n*=35). (**b**) Higher omentin levels were observed in mild ARDS patients. (**c**) Higher omentin levels were maintained in survivors. The omentin levels were not correlated with the duration of ventilation (**d**), the length of RICU stay (**e**) and the length of hospital stay (**f**) in patients with ARDS. The omentin levels negatively correlated with white blood cells (**g**) and procalcitonin (**h**) in patients with ARDS. The data are presented as the mean±S.D. The lines indicate the mean value and the error bars indicate the S.D. *P<0.05

First, we examined whether the circulating omentin levels are associated with ARDS. In total, 38 adult patients with ARDS and 35 healthy controls were enrolled in the study, with no significant statistical differences in the basic demographics (Supplementary Table S1). Compared with the healthy controls, the plasma omentin levels were lower in patients with ARDS (Figure 1a). Compared with the healthy controls, the plasma omentin levels were lower in patients with ARDS (Figure 1a). Moreover, higher omentin levels were observed in mild ARDS patients (Figure 1b). In addition, we also compared the omentin levels of 21 survivors and 17 nonsurvivors (Supplementary Table S2). Compared with the nonsurvivors, the survivors maintained higher concentrations of omentin (Figure 1c). However, there were no correlations between the circulating omentin and the duration of ventilation, the length of respiratory intensive care unit (RICU) stay or hospital stay in patients with ARDS (Figure 1d–f). Thereafter, we further evaluated whether the circulating omentin levels are associated with the inflammatory response in patients with ARDS. The circulating omentin concentrations negatively correlated with white blood cells (WBCs) and procalcitonin (PCT) levels in patients with ARDS (Figure 1g and h). This work indicates the clinical role of omentin in ARDS severity and inflammation, suggesting omentin as a potential target for the future therapeutic strategies.

### Omentin suppresses pulmonary inflammation in mouse lung tissue and ECs

**Figure 2:**
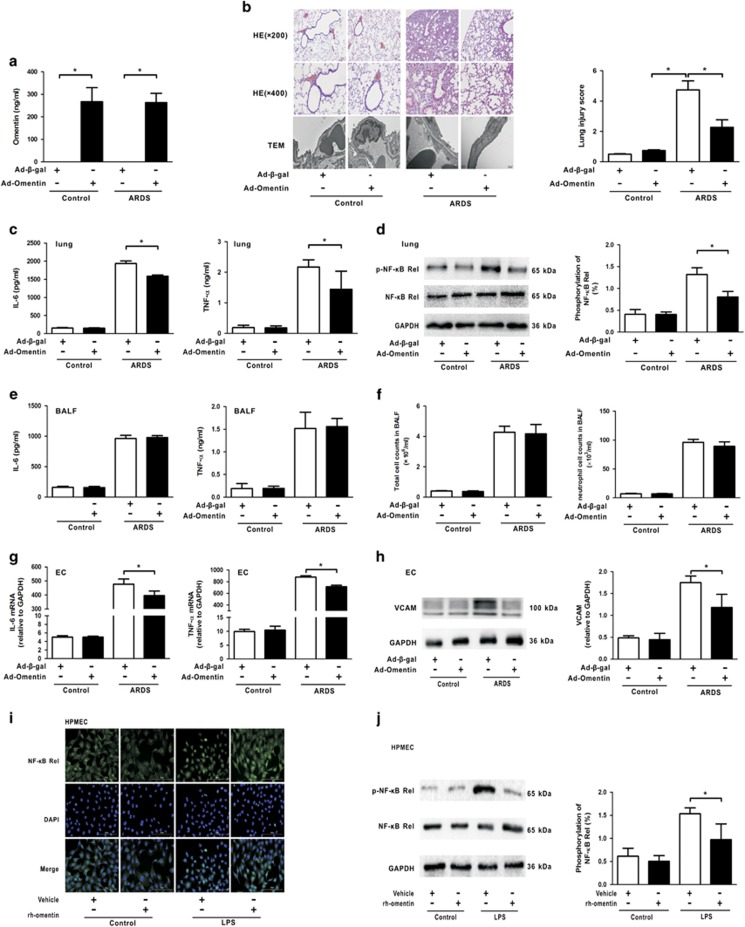
Omentin suppresses pulmonary inflammation in mouse lung tissue and pulmonary endothelial cells (ECs). Ad-omentin or Ad-*β*-gal as control (3 × 107 PFU total per mouse) was injected into the internal jugular vein of mice for 3 days. (**a**) On the third day following adenovirous injection, the circulating omentin levels in Ad-omentin-treated mice increased to 267.8±42.7 ng/ml. Mice were subjected to intratracheal injection with LPS (5 mg/kg) or PBS. At 4 h after LPS injection, lung lobes were isolated to evaluate the histology and ultrastructural pathological damages. (**b**) H&E staining (magnification, × 200 and × 400) demonstrated that Ad-omentin significantly attenuated LPS-induced lung histopathologic alterations, including neutrophil infiltration, perivascular exudates, thickened alveolar septum, intra-alveolar and interstitial edema fluid, and hemorrhage. Lung injury scores were utilized for the quantitative analysis of lung histopathologic damage. Transmission electron microscopy showed that LPS-induced ultrastructural injuries in pulmonary ECs were significantly alleviated by Ad-omentin (*n*=6 independent mice from each group assayed in triplicate). At 4 h after LPS instillation, lung tissue and bronchoalveolar lavage fluid (BALF) were collected. (**c**) In lung tissue, Ad-omentin diminished the levels of IL-6 and TNF-*α* after LPS instillation (*n*=6 independent mice from each group assayed in triplicate). (**d**) In lung tissue, Ad-omentin inhibited the phosphorylation of the NF-*κ*B Rel subunit after LPS instillation (*n*=6 independent mice from each group analyzed in triplicate). (**e**) In BALF, there were no significant differences in the levels of IL-6 and TNF-*α* (*n*=6 independent mice from each group analyzed in triplicate). (**f**) In BALF, there were no significant differences in the total cell and neutrophil counts (*n*=6 independent mice from each group analyzed in triplicate). (**g**) Quanitative RT-PCR analysis showed the gene levels of TNF-*α* and IL-6, normalized to the mRNA expression of GAPDH, were reduced in pulmonary ECs isolated from Ad-omentin-pretreated mice after LPS instillation (*n*=6 independent mice from each group analyzed in triplicate). (**h**) Western blot analysis showed that the protein levels of VCAM were decreased in pulmonary ECs isolated from Ad-omentin-pretreated mice after LPS instillation (*n*=6 independent mice from each group analyzed in triplicate). The relative abundances of protein bands were quantified by measuring the corresponding band intensities; the relative values are expressed normalized to GAPDH signals as shown in the bar graphs. (**i**) Administration of rh-omentin diminished the nuclear translocation of the NF-*κ*B Rel subunit in HPMECs 2 h after LPS insult (*n*=3 independent cultures from each group assayed in triplicate, magnification, × 400). (**j**) Western blot analysis showed that the administration of rh-omentin reduced the phosphorylation of the NF-*κ*B Rel subunit in HPMECs 2 h after LPS insult (*n*=3 independent cultures from each group assayed in triplicate). The relative abundances of protein bands were quantified by measuring the corresponding band intensities; the phosphorylation levels of NF-*κ*B Rel subunit are expressed normalized to the total NF-*κ*B Rel subunit signals as shown in the bar graphs. The data are presented as the mean±S.D. *P<0.05

To assess the effects of increased circulating omentin levels on pulmonary injury response, lung histological examination and ultrastructure pathological examination were performed. Mice were systemically treated with adenoviral vector expressing omentin (Ad-omentin) or adenoviral vectors expressing *β*-galactosidase (Ad-*β*-gal) as control 3 days before being intratracheally (i.t.) injected with lipopolysaccharide (LPS) to develop ARDS. On the third day following adenoviral vectors injection, the circulating omentin level reached 267.8±42.7 ng/ml in Ad-omentin-treated mice, similar to the circulating omentin level in healthy human controls in our clinical study (Figure 2a). Compared with the control group, the mice developed obvious ARDS 4 h after being i.t. injected with LPS, as indicated by histopathologic damage in the lungs, including thickening alveolar septum, perivascular space edema, neutrophil infiltration, proteinaceous edema fluid, patchy hemorrhage areas and severe ultrastructure damage in pulmonary ECs. These pathological changes were attenuated by the pre-treatment of Ad-omentin (Figure 2b). Lung injury in ARDS is coupled to an exaggerated inflammatory response; therefore, we assessed the effects of omentin on the pulmonary inflammatory response 4 h after LPS challenge. Consistent with the lung injury assessment results, the levels of inflammatory cytokines IL-6 and TNF-*α* (Figure 2c), and the phosphorylation of the NF-*κ*B Rel subunit (Figure 2d), a pivotal inflammatory mediator, in lung tissue were diminished in Ad-omentin-pretreated mice compared with Ad-*β*-gal-pretreated mice after LPS instillation. However, no differences in inflammatory cytokine concentrations (Figure 2e) or cell counts (Figure 2f) were observed in the bronchoalveolar lavage fluid (BALF), suggesting that Ad-omentin-mediated mitigation of lung injury was associated with its anti-inflammatory effects outside the pulmonary alveolus. ECs, another crucial target for ARDS, are reported to benefit from omentin.^30, 31^ Therefore, the gene expression of inflammatory cytokines and the protein expression of the adhesion marker VCAM were analyzed in primary ECs isolated from mouse lung. The levels of IL-6 and TNF-*α* (Figure 2g), and VCAM (Figure 2h) were reduced in primary ECs isolated from the lungs of Ad-omentin-pretreated mice after LPS instillation. The observed anti-inflammatory effect of omentin on ECs was further confirmed by examining NF-*κ*B activation, and the results demonstrated that recombinant human omentin (rh-omentin) diminished the nuclear translocation (Figure 2i) and phosphorylation of the NF-*κ*B Rel subunit (Figure 2j) in human pulmonary microvascular ECs (HPMECs) 2 h after LPS insult. Taken together, these findings indicate that pulmonary ECs are the main target for omentin's anti-inflammatory effect.

### Omentin promotes pulmonary endothelial barrier function after LPS insult *in vivo* and *in vitro*

**Figure 3:**
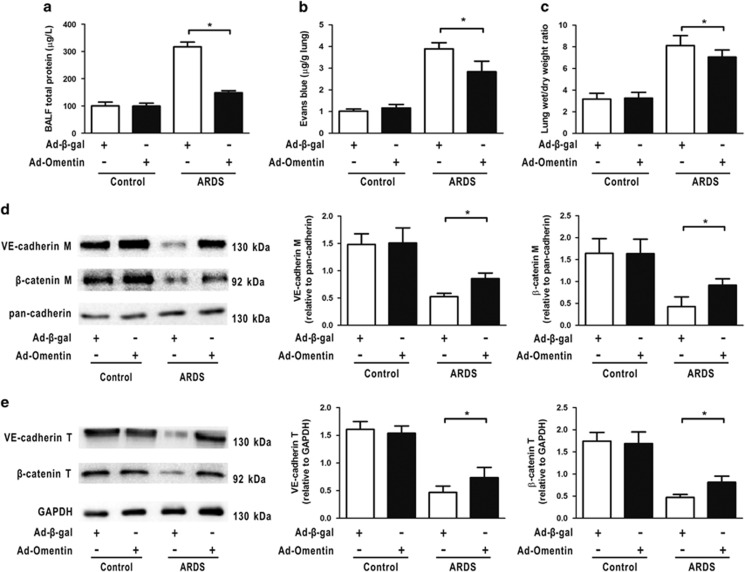
Omentin promotes pulmonary endothelial barrier function after LPS insult in mice. Mice were systemically pre-treated with Ad-omentin or Ad-*β*-gal as a control (3 × 10^7^ PFU total per mouse) and subjected to intratracheal injection with LPS (5 mg/kg) or PBS. Ad-omentin significantly reduced the total BALF protein concentrations (**a**), EBDA extravasation (**b**) and W/D ratios (**c**) in a murine model of ARDS (*n*=6 independent mice from each group analyzed in triplicate). (**d**) Western blot analysis showed that Ad-omentin significantly reversed LPS-induced reduction in the membrance protein expression of VE-cadherin and *β*-catenin in mouse lung tissue after LPS instillation (*n*=6 independent mice from each group analyzed in triplicate). The relative abundances of protein bands were quantified by measuring the corresponding band intensities; the relative values are expressed normalized to pan-cadherin signals as shown in the bar graphs. (**e**) Western blot analysis showed that Ad-omentin significantly reversed LPS-induced reduction in the total protein expression of VE-cadherin and *β*-catenin in mouse lung tissue after LPS instillation (*n*=6 independent mice from each group analyzed in triplicate). The relative abundances of protein bands were quantified by measuring the corresponding band intensities; the relative values are expressed normalized to GAPDH signals as shown in the bar graphs.The data are presented as the mean±S.D. *P<0.05

Increased pulmonary microvascular permeability is a cardinal feature of ARDS. Therefore, to address whether omentin could rescue capillary endothelial barrier function *in vivo*, total BALF protein concentrations, evans blue-dyed albumin (EBDA) extravasation and wet/dry (W/D) ratios were analyzed. We found that histological pulmonary damage was associated with the capillary leakage exacerbation after LPS instillation, as manifested by increases in BALF protein concentrations (Figure 3a), EBDA extravasation (Figure 3b) and W/D ratios (Figure 3c) at 4 h after LPS, which was the period associated with the histological onset of lung injury in mice. Significant reduction in capillary leakage (Figures 3a–c), accompanied by enhancements in the membrane and total protein expression of AJs (*β*-catenin and VE-cadherin, Figures 3d and e) were observed in the Ad-omentin-pretreated mice compared with those pre-treated with LPS alone. Our data demonstrated that omentin significantly attenuated LPS-challenged pulmonary microvascular leakage and increased AJs protein expression in a murine model of ARDS. To further confirm omentin's ability to mitigate LPS-induced EC hyperpermeability *in vitro*, HPMECs were cultured in the presence or absence of rh-omentin for 24 h, and the influx of FITC–dextran was measured. Treatment with a physiological concentration of rh-omentin (300 ng/ml) prevented the LPS-induced increase in the influx of FITC–dextran (Supplementary Figure S1), suggesting that omentin restored EC barrier dysfunction *in vitro*.

### Omentin improves pulmonary EC survival and differentiation after LPS insult *in vitro*

**Figure 4:**
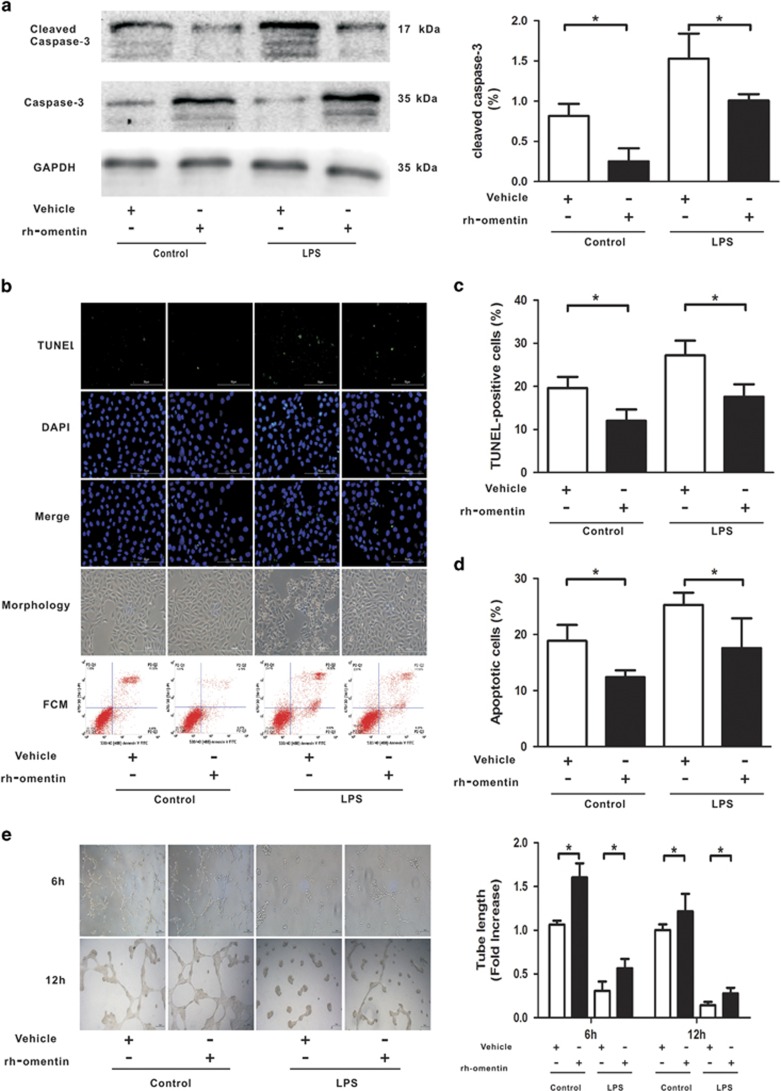
Omentin improves pulmonary EC survival and differentiation after LPS insult in HPMECs. HPMECs were cultured with rh-omentin (300 ng/ml) or PBS for 24 h and exposed to either PBS or LPS (100 ng/ml) for 2 h. (**a**) Western blot analysis showed that rh-omentin reduced the level of cleaved caspase-3 in cultured HPMECs under both unstressed and LPS insult conditions (*n*=5 independent cultures from each group assayed in triplicate). The relative abundances of protein bands were quantified by measuring the corresponding band intensities; the relative values are expressed normalized to GAPDH signals as shown in the bar graphs. (**b**) TdT-mediated dUTP nick end labeling (TUNEL) staining and flow cytometry showed that rh-omentin diminished the ratios of TUNEL-positive cells and apoptotic cells under both unstressed and LPS insult conditions (*n*=3 independent cultures from each group assayed in triplicate, magnification, × 400). (**c**) Quantitative analyzes of TUNEL-positive HPMECs are shown in the bar graphs. (**d**) Quantitative analyzes of the ratios of apoptotic cells are shown in the bar graphs. (**e**) Endothelial cell tube formation assay demonstrated that rh-omentin promoted EC differentiation under LPS insult conditions, as evident by the increased levels of tube length (*n*=3 independent cultures from each group assayed in triplicate). HPMECs were grown on 48-well plates pre-coated with Matrigel (BD, NJ, USA) at a concentration of 4 × 10^4^ cells per 200 *μ*l. After cell pellets were added to the EGM of corresponding interventions, they were cultured in an incubator at 37 °C with 5% CO_2_ for 12 h and then examined under a phase-contrast microscope (BX51 Olympus, Japan) with a × 10 objective. Representative photomicrographs on the left indicate HPMECs differatation into tubes. Quantitative analyzes of tube length are shown in the bar graphs.The data are presented as the mean±S.D. *P<0.05

Focusing primarily on the pulmonary EC barrier, we further investigated the beneficial effects of omentin on pulmonary ECs at the cellular level. HPMECs were subjected to LPS stimulation in the presence of rh-omentin protein or vehicle. First, we examined the effects of omentin on the survival and apoptosis of pulmonary ECs after exposure to LPS. Cell counting kit 8 (CCK-8), cleaved caspase-3, TdT-mediated dUTP nick end labeling (TUNEL) staining and flow cytometry (FCM) analyzes demonstrated that omentin significantly promoted pulmonary EC survival and suppressed pulmonary EC apoptosis, as evident by the enhanced cell survival (Supplementary Figure S2) and reduced levels of cleaved caspase-3 (Figure 4a), accompanied by lower ratios of TUNEL-positive cells (Figures 4b and c) and apoptotic cells (Figures 4b and d) under both unstressed and LPS insult conditions, suggesting a pro-survival property of omentin. Next, to examine whether omentin modulates pulmonary EC differentiation, HPMECs plated on a Matrigel matrix were treated with rh-omentin protein or vehicle 30 min before LPS exposure. Quantitative analysis of EC differentiation into vascular-like tubes was performed at the indicated time point, demonstrating that omentin significantly increased the tubes and branches relative to control cultures, indicating a pro-angiogenic function of omentin (Figure 4e).

### Omentin stabilizes pulmonary EC AJs and actin cytoskeleton after LPS insult *in vitro*

**Figure 5:**
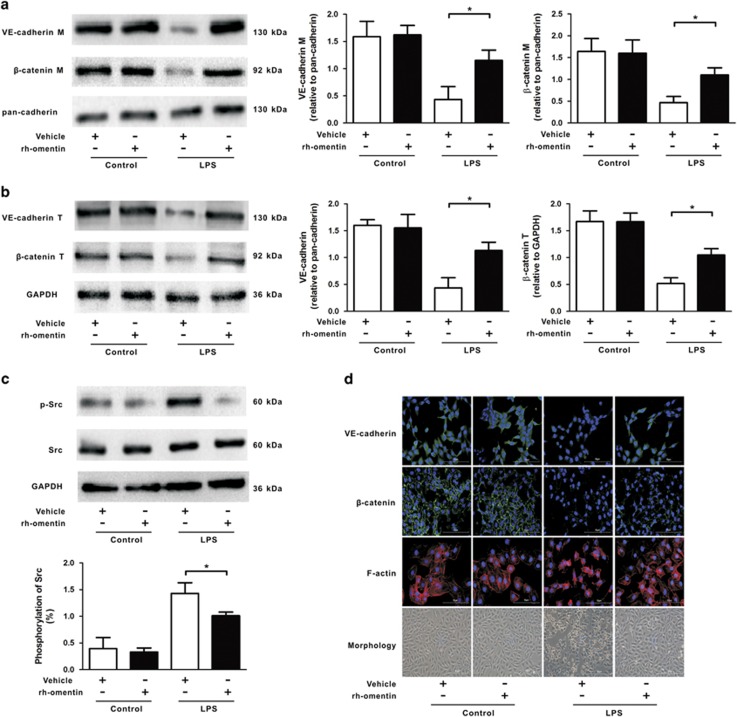
Omentin stabilizes pulmonary EC AJs and actin cytoskeleton after LPS insult in HPMECs. HPMECs were cultured with rh-omentin (300 ng/ml) or PBS for 24 h and exposed to either PBS or LPS (100 ng/ml) for 2 h. (**a**) Western blot analysis showed that rh-omentin enhanced the membrane protein expression of VE-cadherin and *β*-catenin under LPS insult conditions (*n*=5 independent cultures from each group assayed in triplicate). The relative abundances of protein bands were quantified by measuring the corresponding band intensities; the relative values are expressed normalized to pan-cadherin signals as shown in the bar graphs. (**b**) Western blot analysis showed that rh-omentin enhanced the total protein expression of VE-cadherin and *β*-catenin under LPS insult conditions (*n*=5 independent cultures from each group assayed in triplicate). The relative abundances of protein bands were quantified by measuring the corresponding band intensities; the relative values are expressed normalized to GAPDH signals as shown in the bar graphs. (**c**) Western blot analysis showed that rh-omentin diminished the level of phosphorylated Scr under LPS insult conditions (*n*=5 independent cultures from each group assayed in triplicate). The relative abundances of protein bands were quantified by measuring the corresponding band intensities; the relative values of the phosphorylated Scr are expressed normalized to the total Scr signals, as shown in the bar graphs. (**d**) Immunofluorescence staining showed that rh-omentin reversed the LPS-induced decrease in the expression of *β*-catenin and VE-cadherin. Phalloidin staining showed that rh-omentin inhibited cell retraction, F-actin reorganization and stress fiber formation, which was induced by LPS challenge. Cell morphology analyzes showed a transition from the flattened quiescent to rounded active endothelial phenotype under LPS insult conditions, which was reversed by rh-omentin (*n*=3 independent cultures from each group assayed in triplicate, magnification, × 400). The data are presented as the mean±S.D. *P<0.05

The stabilization of endothelial AJs and actin cytoskeleton is essential for a restrictive pulmonary EC barrier and lung endothelium permeability can increase because of alternations in AJs and endothelial cytoskeleton. Therefore, to address the effects of omentin on vascular homeostasis, the expression of AJs (*β*-catenin and VE-cadherin) was measured by western blot (WB) and immunofluorescence (IF) staining, the assembly of actin cytoskeleton was assessed by phalloidine staining, and the activation of Src was determined by WB in HPMECs. As expected, LPS-insulted HPMECs exhibited a reduced expression level of AJs (Figures 5a and b) and an elevated level of phosphorylated Scr (Figure 5c), as well as a transition from the flattened quiescent to rounded active endothelial phenotype, which was further confirmed by phalloidin staining showing cell retraction, F-actin reorganization and stress fiber formation compared with control cells (Figure 5d). Administration of rh-omentin reversed the deleterious effects of LPS on pulmonary ECs, as demonstrated by the enhanced membrane and total abundance of AJs protein (Figures 5a and b), as well as the diminished phosphorylated Scr levels (Figure 5c) and a well-arranged cortical actin rim (Figure 5d). In the unchallenged state, AJ expression and actin cytoskeleton distribution in omentin-treated HPMECs were not altered compared with those of untreated controls (Figure 5), indicating that in contrast to its pro-survival property, omentin could only exert beneficial effects on pulmonary EC barrier integrity in the LPS state. Taken together, these results indicate that omentin reinforces the pulmonary EC barrier by stabilizing AJs and the actin cytoskeleton.

### Omentin activates Akt-related signaling pathways *in vivo* and *in vitro*

**Figure 6:**
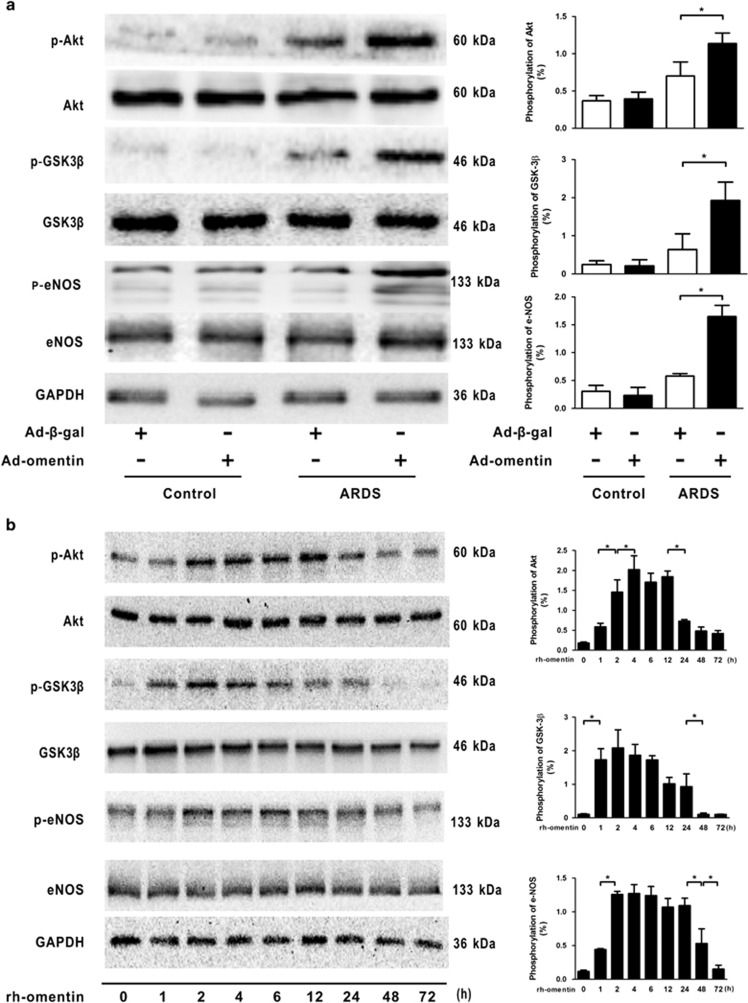
Omentin activates Akt-related signaling pathways in mice and in HPMECs. (**a**) Mice were systemically pre-treated with Ad-omentin or Ad-*β*-gal as a control (3 × 10^7^ PFU total per mouse) and subjected to intratracheal injection with LPS (5 mg/kg) or PBS. Western blot analysis demonstrated that Ad-omentin enhanced the phosphorylation of Akt (p-Akt), GSK-3*β* (p-GSK-3*β*) and eNOS (p-eNOS) in mouse lungs subjected to LPS (*n*=6 independent mice from each group analyzed in triplicate). (**b**) HPMECs were cultured with rh-omentin (300 ng/ml) or PBS for 24 h and exposed to either PBS or LPS (100 ng/ml) for 2 h. Western blot analysis showed the time-dependent changes in p-Akt, p-GSK-3*β* and p-eNOS in HPMECs, following rh-omentin stimulation (*n*=5 independent cultures from each group assayed in triplicate). The relative abundances of protein bands were quantified by measuring the corresponding band intensities; the relative phosphorylation levels of protein are expressed normalized to the total protein signals, as shown in the bar graphs. The data are presented as the mean±S.D. *P<0.05

The PI3K/Akt signaling pathway acts as a compensatory regulator of ARDS through its inflammatory and angiogenic responses to multiple growth factors. Therefore, to assess the effects of omentin on the activation of the Akt-related signaling *in vivo* and *in vitro*, the phosphorylation of Akt and eNOS was assessed by WB. We observed that p-Akt and p-eNOS levels were low under non-stressed conditions, but were increased in LPS-challenged ARDS mouse lungs and HPMECs, although the differences were not statistically significant between the two groups, suggesting an endogenous negative feedback mechanism of the PI3K/Akt pathway for LPS. Notably, Ad-omentin administration enhanced the phosphorylation of Akt and GSK-3*β*, a direct target of Akt in mouse lungs subjected to LPS (Figure 6a), indicating that omentin is a stimulatory factor for Akt-related signaling pathways. At the cellular level, rh-omentin stimulated the phosphorylation of Akt and GSK-3*β* in a time-dependent manner (Figure 6b). As a key downstream target of Akt signaling, eNOS is involved in the angiogenic response and survival activity of several growth factors in ECs. Accordingly, the eNOS phosphorylation levels *in vivo* and *in vitro* were further assessed by WB. Compared with that of vehicle-pretreated mice, pretreatment with Ad-omentin enhanced LPS-induced upregulation of eNOS phosphorylation after LPS instillation (Figure 6a). In HPMECs, rh-omentin stimulated eNOS phosphorylation in a time-dependent manner, with maximal induction occurring at 120 min (Figure 6b).

### Akt/eNOS signaling contributes to omentin-mediated protection of the pulmonary endothelial barrier *in vivo* and *in vitro*

**Figure 7:**
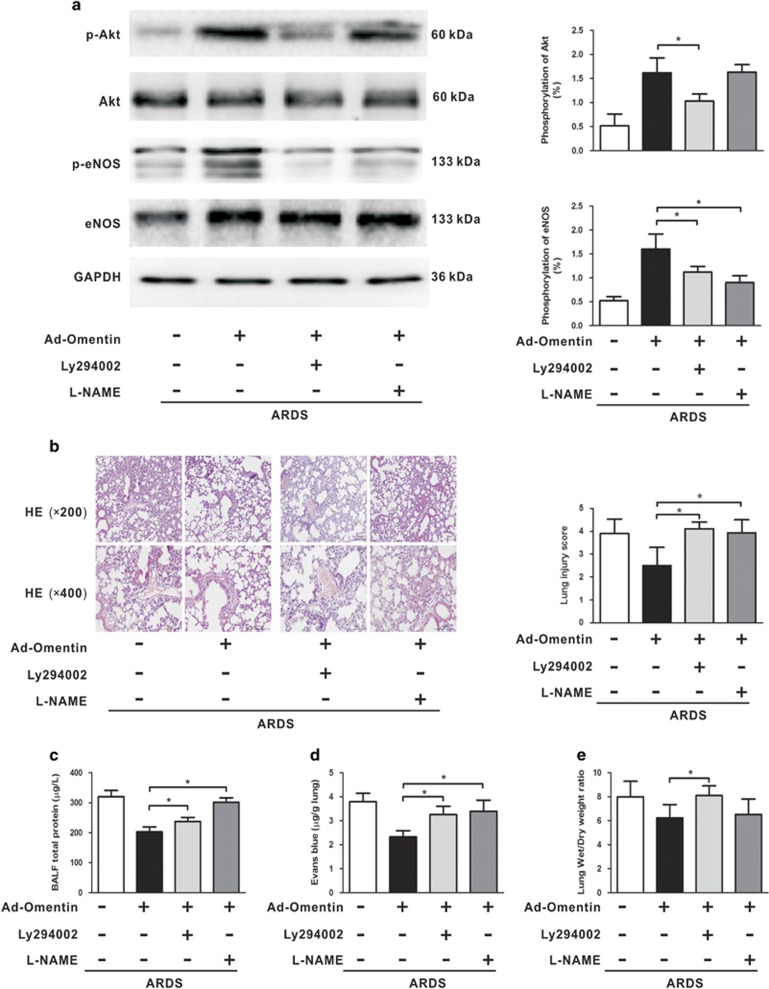
Akt/eNOS activation participates in omentin-mediated protection of the pulmonary endothelial barrier in mice. Mice were systemically pre-treated with Ad-omentin or Ad-*β*-gal as control (3 × 10^7^ PFU total per mouse) and subjected to intratracheally injection with LPS (5 mg/kg) or PBS. The Akt inhibitor LY294002 (40 mg/kg, dissolved in dimethyl sulfoxide (DMSO)), eNOS inhibitor L-NAME (100 mg/kg) or vehicle (10% DMSO in PBS) was intraperitoneally injected into mice 1 h before LPS or PBS instillation. (**a**) Western blot analysis showed that LY294002 inhibited the phosphorylation of Akt and eNOS in LPS-induced ARDS mice (*n*=6 independent mice from each group analyzed in triplicate). The relative abundances of protein bands were quantified by measuring the corresponding band intensities; the relative phosphorylation levels of protein are expressed normalized to the total protein signals, as shown in the bar graphs. (**b**) H&E staining showed that LY294002 and L-NAME aggravated histological injury in LPS-induced ARDS mice (*n*=6 independent mice from each group analyzed in triplicate, magnification, × 200 and × 400). Lung injury scores were utilized for quantitative analysis of lung histopathologic damage. (**c**) Bar graphs showed that LY294002 and L-NAME increased BALF protein levels and exacerbated EBDA extravasation in LPS-induced ARDS mice (*n*=6 independent mice from each group analyzed in triplicate). The data are presented as the mean±S.D. *P<0.05

**Figure 8:**
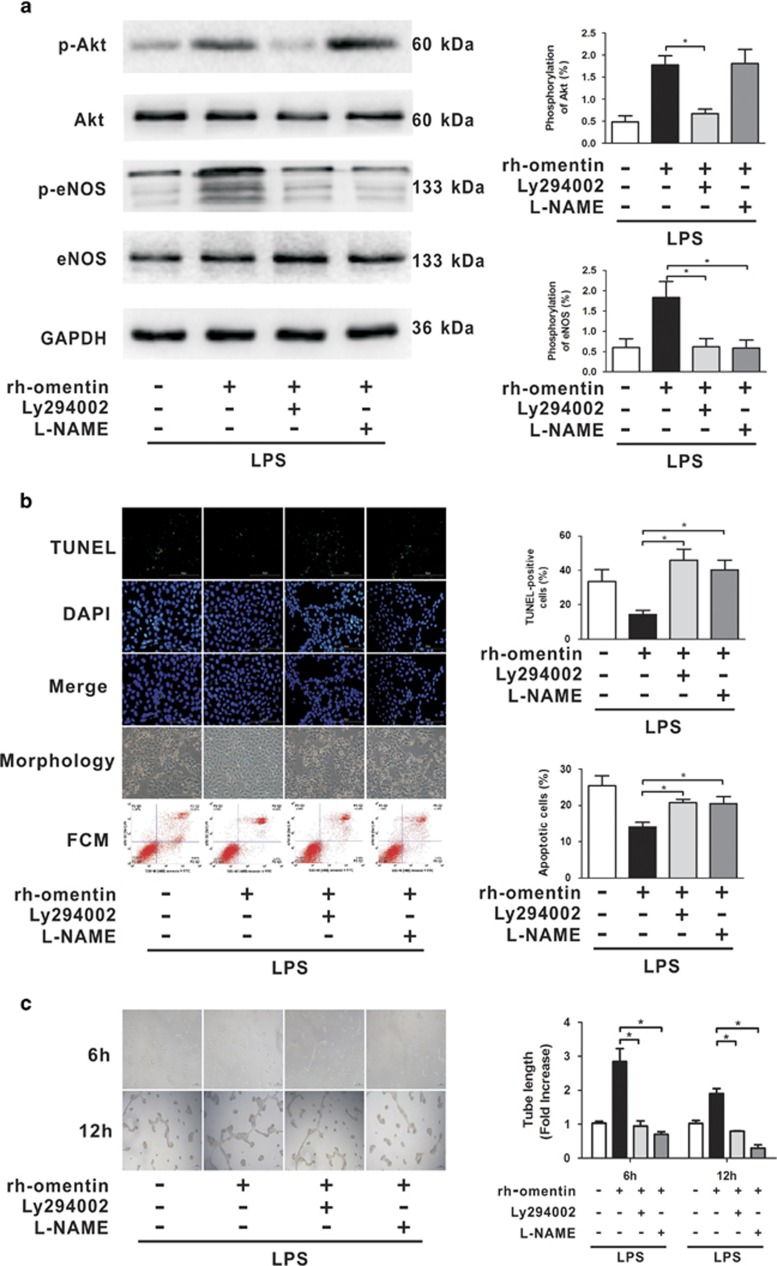
Akt/eNOS activation contributes to omentin's promoting effects on the pulmonary EC barrier in HPMECs. HPMECs were pretreated with LY294002 (50 μM), L-NAME (1 mM) or vehicle (10% DMSO in PBS) 60 min before LPS and cultured with rh-omentin (300 ng/ml) or PBS for 24 h followed by exposure to LPS (100 ng/ml) for 2 h. (**a**) Western blot analysis showed that LY294002 inhibited rh-omentin-induced phosphorylation of Akt and eNOS, and that L-NAME blocked rh-omentin-stimulated phosphorylation of eNOS with no effects on Akt phosphorylation (*n*=5 independent cultures from each group assayed in triplicate). The relative abundances of protein bands were quantified by measuring the corresponding band intensities; the relative phosphorylation levels of protein are expressed normalized to the total protein signals, as shown in the bar graphs. (**b**) TUNEL staining, FCM and cell morphology analyzes demonstrated that LY294002 and L-NAME suppressed rh-omentin stimulated anti-apoptotic effects on pulmonary EC apoptosis as evident by elevated ratios of TUNEL-positive cells and apoptotic cells (*n*=3 independent cultures from each group assayed in triplicate, magnification, × 400)). (**c**) Endothelial cell tube formation assay demonstrated that LY294002 and L-NAME blocked rh-omentin-promoted EC differentiation under LPS insult conditions, as evidenced by decreased levels of tube length (*n*=3 independent cultures from each group assayed in triplicate, magnification, × 100). Representative photomicrographs on the left indicate HPMECs differatation into tubes. Quantitative analyzes of tube length are shown in the bar graphs.The data are presented as the mean±S.D. *P<0.05

Mice were pretreated with the Akt inhibitor LY294002 or with the eNOS inhibitor L-NAME 1 h before LPS insult to confirm the involvement of Akt/eNOS signaling in omentin-mediated protection against LPS-induced ARDS *in vivo*. We found that pretreatment with LY294002 inhibited the Ad-omentn-stimulated phosphorylation of Akt and eNOS (Figure 7a), and that LY294002 and L-NAME aggravated histological injury (Figure 7b), increased BALF protein levels (Figure 7c) and exacerbated EBDA extravasation (Figure 7d) in Ad-omentn-pretreated mice after LPS challenge. Thus, these findings suggest that omentin exerts endothelial-protective effects on LPS-induced ARDS mice at least partly through the Akt/eNOS signaling pathway. To further confirm whether Akt/eNOS activation participates in omentin's promoting effects on the pulmonary ECs *in vitro*, HPMECs were treated with LY294002 or L-NAME 1 h before LPS insult. As expected, LY294002 inhibited the phosphorylation of Akt and eNOS (Figure 8a), and both LY294002 and L-NAME reversed rh-omentin's stimulatory effects on cell survival (Figure 8b) and differentiation (Figure 8c) under LPS challenge. Taken together, these findings indicate that omentin exerts promoting effects on pulmonary ECs at least in part through the Akt/eNOS signaling.

### One-shot treatment with rh-omentin protein alleviates pulmonary inflammation and endothelial injury after LPS-induced ARDS in mice

To explore the therapeutic potential of omentin, mice were administered rh-omentin protein 4 h after LPS insult. Lung injury, which was evaluated by histological and ultrastructural pathological examination 24 h after LPS instillation, was mitigated in the rh-omentin-treated group compared with the control group (Supplementary Figures S3A and B). Moreover, rh-omentin-treated mice exhibited decreased pulmonary inflammation,as evidenced by decreased levels of the pro-inflammatory cytokines IL-6 (Supplementary Figure S3C) and TNF-*α* (Supplementary Figure S3D), and of the adhesion markers VCAM and phosphorylated NF-*κ*B Rel in the lungs (Supplementary Figure S3E). Pulmonary endothelial barrier function and integrity, which were assessed by BALF protein concentrations (Supplementary Figure S3F), EBDA extravasation (Supplementary Figure S3G), the W/D ratio (Supplementary Figure S3H) and AJ expression (Supplementary Fig. S3I), were restored. In addition, omentin administration resulted in an increase in the phosphorylation of Akt and eNOS in the lungs (Supplementary Figure S3J). Collectively, these findings suggest that the therapeutic potential of omentin for treating ARDS functions at least in part by activating the Akt/eNOS pathway.

## Discussion

The present study is the first to demonstrate that omentin protects against LPS-induced ARDS by limiting the inflammatory response and promoting the pulmonary endothelial barrier. Clinically, a decreased level of circulating omentin negatively correlated with WBC and PCT levels in patients with ARDS. Systemic delivery of Ad-omentin exerted beneficial effects on the pulmonary endothelium by limiting the pulmonary inflammatory response and endothelial barrier injury in murine models of ARDS. In HPMECs, treatment with rh-omentin protein resulted in the enhancement of EC survival and differentiation and of AJ expression, as well as attenuation of the inflammatory response, cell apoptosis and cytoskeleton rearrangement. Further mechanistic study indicated that the Akt/eNOS signaling pathway at least partially contributed to these favorable effects of omentin. Therapeutic treatment with rh-omentin protein was also effective in suppressing endothelial inflammation and reinforcing the EC barrier in mice. Collectively, these data suggest that therapeutic approaches to restore omentin levels may be valuable for the prevention or treatment of ARDS.

ARDS is pathologically characterized by an uncontrolled inflammatory response and widespread alveolar-endothelial injury.^1^ As a novel biomarker for endothelial damage and obesity-related vascular diseases in human study,^24, 26, 28^ omentin has also been shown to be involved in modulating inflammation, apoptosis and angiogenesis in experimental studies.^37, 38, 39^ All these underlying mechanisms were implicated in the pathogenesis of ARDS; the anti-inflammatory and endothelial-protective properties of omentin were elucidated in our present study.

Inflammation plays a pivotal role in the initiation and progression of ARDS.^6^ Although omentin has been indicated as an anti-inflammatory adipokine in the pathogenesis of various obesity-related disorders, its specific role and underlying mechanism in ARDS remain unclear.^14, 15, 16, 22^ In agreement with the previous findings that omentin played an anti-inflammatory role in EC cells through inhibiting TNF-*α*-induced VCAM expression by blocking the NF-*κ*B pathway,^30^ our present study demonstrated that omentin suppressed the inflammatory response to LPS by targeting the lung EC barrier. Although our study focused on the direct effects of omentin on the endothelium, we cannot exclude its possible direct effects on lung immune cells or its indirect effects on the production of chemotactic factors or other cytokines, which may subsequently affect neutrophil recruitment. Therefore, additional studies are needed to establish the role of omentin in these effects.

The importance of the pulmonary endothelial barrier in ARDS has been well documented.^4^ The EC barrier consists of the cytoskeleton, AJs and tight junctions (TJs). In the current concept, TJs play a minor role in the barrier function of the pulmonary endothelium, whereas AJs play a vital role in maintaining the connection with adjacent ECs through extracellular binding of VE-cadherin and catenins, which are further stabilized by intracellular linkages with the actin cytoskeleton, thereby regulating pulmonary permeability and fluid homeostasis.^4, 5, 43, 44^ We found that increased pulmonary microvascular permeability in ARDS was effectively alleviated by the systemic administration of Ad-omentin, indicating omentin's endothelial-promoting property *in vivo*. Further *in vitro* research at the cellular level supports this favorable property. Consistent with our notion, experimental studies reported that omentin improves EC function and revascularization progress in mouse muscle tissue and in cultured ECs.^37^ In human research, circulating omentin has been regarded as a useful marker of endothelia function.^24^ The pro-survival property of omentin has also been demonstrated in varying tissue and cell types under conditions of multiple injurious stimuli.^37, 38, 39, 41^ Although we acknowledged the protective effect of omentin on the endothelium, the function of the lung epithelium cannot be ignored. Further studies assessing the effects of omentin on lung epithelial cells are needed.

Akt-related signaling pathways are known to function as compensatory mechanisms by regulating multiple cellular functions.^35, 36, 45^ Moreover, omentin-stimulated the activation of Akt signaling has been reported to play a crucial role in the response to injurious stimulator via promoting cell survival and revascularization in muscle and heart tissues.^37, 38^ Therefore, to determine the underlying mechanism of omentin-mediated protection against LPS-induced ARDS, we further investigated the contribution of Akt-related signaling *in vivo* and *in vitro*. Here, consistent with the previous findings, treatment of mice and HPMECs with omentin activated Akt-related signaling. However, there were no significant differences in omentin-mediated anti-inflammatory effects when Akt signaling was blocked, suggesting that Akt signaling may not be predominant in omentin-mediated anti-inflammatory effects against LPS-induced ARDS and that the activation of other pathways likely contributes more to these effects.

eNOS, which is downstream of Akt, acts as a crucial regulator of vascular growth and EC function.^46, 47^ Omentin has been reported to promote endothelium-dependent vasodilation in aorta isolated from rat and to reduce cytokine-induced inflammatory responses in cultured ECs; these effects were prevented by a NOS inhibitor.^29, 31^ Moreover, omentin promotes EC differentiation and survival by activating Akt/eNOS signaling in both ischemic muscles and cultured ECs.^37^ These data suggest that eNOS may act as an angiogenic mediator in omentin-mediated protective effects on the vasculature. In agreement with the previous findings, we demonstrated that omentin exerted promoting effects on pulmonary endothelial barrier at least partly via an Akt/eNOS-dependent mechanism. However, the signaling pathways mediated the protective effects of omentin are complex and never exclusive. Other inflammation-related signaling pathway, especially those inducing the activation of NF-kB such as SAPK/ JNK, p38 MAPK and ERK1/2 pathways, are highly noticeable for their involvement in the regulation of inflammatory response. Notably, AMPK functions upstream of PI3K/Akt signaling in ECs.^48^ The roles of AMPK have also been demonstrated to meditate the effects of omentin on blood flow and EC via eNOS, which will be investigated in our further studies.

However, considering that the specific receptor of omentin has not been identified and that omentin acts as a pleiotropic adipokine, further studies are necessary to define the specific and overlapping contributions of other signaling pathways that mediate the protective effects of omentin in ARDS. Moreover, adipose tissue is considered a significant endocrine organ that is capable of crosstalk with peripheral organs through various multi-functional adipokines; thus future prospective clinical studies are needed to confirm the association between various adipokines and pathogenesis of ARDS. Futhermore, the protective effects of omentin on pulmonary endothelium in ARDS are not exclusive. Contributions of other adipokines, especially those exert anti-inflammatory and vascular-protective effects such as omentin, adipolin, CTRP9 and their homeostasis must be elucidated in future studies.

## Conclusion

Collectively, our study demonstrates that omentin can exert protective effects on the pulmonary endothelial barrier by suppressing the inflammatory response, promoting survival and differentiation, and stabilizing AJ expression and the actin cytoskeleton of ECs, thus ameliorating LPS-induced ARDS, which may suggest a potential therapeutic intervention for patients with ARDS in clinical practice.

## Materials and Methods

### Clinical study and data collection

We enrolled 38 critically ill adult patients with ARDS addmitted to the RICU between March 2014 and March 2015 at the Second Affiliated Hospital of Chongqing Medical University and were categorized on the day of ARDS diagnosis based on their PaO_2_/FiO_2_ ratio into mild (200<PaO_2_/FiO_2_≤300 mm Hg; *n*=11), moderate (100<PaO_2_/FiO_2_≤200 mm Hg; *n*=19) and severe (PaO_2_/FiO_2_<100 mm Hg; *n*=8) based on the Berlin Definition. Pateints were followed until death in hospital or discharge home with unassisted breathing and then defined as nonsurvivors (*n*=17) or survivors (*n*=21). Demograhic characteristics and clinical data including etiology of ARDS, the acute physiology and chronic health evaluation II (APACHE II) score, PaO_2_/FiO_2_, WBCs, neutrophils, PCT and creative reaction protein concentrations were routinely inspected and recorded at diagnosis. Thereafter, comprehensive clinical outcomes are collected including duration of mechanical ventilation, the length of RICU stay and the length of hospital stay. To analyze the difference in circulating omentin levels between healthy subjects and patients with ARDS, 35 health subjects were recruited as controls. The study protocol was reviewed and approved by the local institutional review board, and written informed consent was obtained from either the patient or each patient's next of kin or legal representative before enrollment. Plasma specimens were obtained from patients with ARDS, as soon as possible after each patient met the defining criteria. In breif, peripheral venous blood samples were centrifuged at 1500 × *g* for 15 min followed by centrifugation at 12 000 × *g* for 2 min to generate platelet-deficient plasma. The deprived plasma samples were immediately stored at −80 °C until use. These samples were used to measure omentin, IL-6 and TNF-*α* concentrations utilizing a commercial enzyme-linked immunosorbent assay (ELISA) kit (R&D, Minneapolis, MN, USA).

### Mouse models of LPS-induced ARDS

Animal experiments were conducted on 10-week-old male C57BL/6 mice of SPF grade (Department of Laboratory Animal Center, Chongqing Medical University). The mice were housed under pathogen-free conditions with a 12 h dark/light cycle and provided food and water *ad libitum*. All animal experimental protocols were implemented according to the instructions of the National Institutes of Health Guide for the Care and Use of Laboratory Animals and approved by the ethics committee of the Second Affiliated Hospital of Chongqing Medical University. The mice were anesthetized with sodium pentobarbital (50 mg/kg intraperitoneally) before the trachea and right internal jugular vein (IJV) were exposed. As described previously, ARDS mouse models were established with one-time i.t. instillation of 5 mg/kg LPS (*Escherichia coli* LPS serotype 0111:B4) in 50 *μ*l of sterile phosphate-buffered saline (PBS) with an 18-Gauge catheter. In total, 3 × 10^7^ PFU of Ad-omentin or Ad-*β*-gal per mouse was injected into the IJV of mice 3 days before LPS or vehicle (PBS) airway installation. On the third day following injection, the circulating omentin level reached 267.8±42.7 ng/ml, similar to the circulating omentin level in healthy human controls in our clinical study. In some experiments, the Akt inhibitor LY294002 (40 mg/kg), eNOS inhibitor L-NAME (100 mg/kg) or vehicle (10% dimethyl sulfoxide in PBS) was intraperitoneally injected into the mice 1 h before LPS or PBS instillation. To explore the therapeutic effects of omentin on the ARDS mouse model, rh-omentin protein (0.15 *μ*g/g per mouse) or PBS was injected into the right IJV 4 h after LPS or vehicle (PBS) airway installation; the mean concentration of circulating human omentin protein was maintained at 253.34±36.5 ng/ml 30 min after rh-omentin administration. At select time points after LPS administration, the mice were killed, and blood, BALF and lung tissue were collected and stored at –80 °C until further analysis.

### Chemicals and reagents

LPS (*E. coli* LPS serotype 0111:B4), sodium pentobarbital, Evans Blue dye, collagenase and trypsin were purchased from Sigma (St. Louis, MO, USA). rh-omentin protein was purchased from GeneTex (Irvine, CA, USA). The following primary antibodies were purchased from Cell Signaling Technology (Danvers, MA, USA): anti-Scr, anti-phospho-Scr (Try416), anti-Akt, anti-phospho-Akt (Ser473), anti-eNOS, anti-phospho-eNOS (Ser1177), anti-NF-*κ*B Rel and anti-phospho-NF-*κ*B Rel (Ser536). Anti-VE-cadherin, anti-pan-cadherin and anti-cleaved caspase-3 antibodies were purchased from Abcam (Cambridge, UK). Anti-GAPDH, anti-CD31, anti-caspase-3, anti-GSK-3*β* and anti-phospho-GSK-3*β* (Ser9) antibodies were purchased from Bioworld Technology (Nanjing, China). Ad-*β*-gal and full-length human omentin (Ad-omentin) were constructed by Genechem (Shanghai, China). Ad-*β*-gal was used as a control.

### Analysis of blood glucose, insulin and lipidemia

Serum blood glucose, insulin, free fatty acid, triglyceride and cholesterol levels were measured using commercially available kits according to the manufacturer's instructions (Biovision, Milpitas, CA, USA).

### H&E staining and lung histology evaluation

Left lung lobes were isolated, fixed in 3.7% paraformaldehyde, embedded in paraffin wax, cut into 5-*μ*m sections and stained with hematoxylin and eosin (H&E). Histological lung injury in each mouse was evaluated in five random fields. The standardized scoring scheme that has been published by the American Thoracic Society was adopted to quantify histological lung injury in the mice.

### Transmission electron microscopy

Fresh lung tissue specimens were isolated, fixed in 2.5% glutaraldehyde, rinsed in phosphate buffer, post-fixed with 1% osmium tetroxide, dehydrated in graded ethyl alcohol, treated with propylene oxide and embedded in epoxy resin. Then, the embedded tissues were thinly sectioned, mounted on copper grids, and stained with uranyl acetate and lead citrate. The images were captured using an electron microscope (H-7500; Hitachi, Tokyo, Japan).

### Analysis of BALF

A trimmed 18-Gauge catheter was inserted into the trachea. A syringe was connected to the catheter, and 1 ml of sterile normal saline was infused into the airway. Four hours after LPS administration, BALF was collected by i.t. instillation of 1 ml of sterile normal saline followed by repeated aspiration three times and centrifugation at 500 × *g* for 10 min at 4 °C. The pellets were resuspended in 50 *μ*l of PBS and stained with Wright-Giemsa (KeyGen Biotech, Nanjing, China). Total and neutrophil cell counts were performed with a hemocytometer in a double-blind manner. The protein concentrations in the BALF supernatants were determined using a bicinchoninic acid protein assay (BCA) kit (KeyGen Biotech).

### ELISA

Aliquots of BALF and lung homogenate supernatant were used to assay the levels of TNF-*α* and IL-6 using the respective commercially available ELISA kits (R&D, Minneapolis, MN, USA) according to the manufacturer's instructions.

### Measurement of EBDA concentrations in the lung

Pulmonary capillary permeability in the lung was assessed by determining EBDA concentrations. The right IJV of mice was injected with EBDA (30 mg/kg). Lungs free of blood were excised, weighed, homogenized in 1 ml of PBS, extracted in 2 ml of formamide (24 h, 60 °C) and centrifuged (5000 × *g*, 30 min, 20 °C). The absorbances of the supernatants, which were measured by spectrophotometry at both 620 and 740 nm, were calculated against a standard curve, normalized as described previously and converted to micrograms of EBDA per gram of lung.

### W/D lung weight ratio

The right upper lung lobes were excised and weighed to determine the wet lung weight. Then, the tissues were dried in an oven at 80 °C for 24 h and then weighed again to calculate the W/D ratios.

### EC culture

Primary lung microvascular ECs were isolated from lung microvessels of male Sprague-Dawley rats (150–200 g) by collagenase and trypsin digestion. Rat lungs were cut into 1-mm^2^ pieces, seeded in gelatin-coated 25-cm^2^ flasks and cultured in DMEM containing 15% FBS, 2% EC growth supplement (ECGS 100 U/ml) and 1% penicillin/streptomycin (P/S) solution at 37 °C in an incubator containing 5% CO_2_ and 95% air. Then, the cells were confirmed based on their morphology and on the expression of a proprietary marker, PECAM-1/CD31.

HPMECs (ScienCell, Carlsbad, CA, USA) were cultured in EC medium (ScienCell) with 10 FBS, 1 ECGS and 1% P/S. For the experiments, the cells between passages 4 and 10 were grown as a monolayer and serum-starved (1% serum) for 6 h before each treatment. Cells were cultured in the presence of 300 ng/ml rh-omentin or vehicle (PBS) for 24 h. In some experiments, the HPMECs were pretreated with LY294002 (50 μM), L-NAME (1 mM) or vehicle (10% DMSO in PBS) for 60 min. Following pretreatment, cells were washed and then exposed to either PBS or LPS at 100 ng/ml for 2 h, the lysates and supernants were collected for later analysis. Cell lysates were collected for later analysis 2 h after LPS insult.

### EC monolayer permeability assay

Permeability was determined based on the paracellular permeability of 70 kDa FITC–dextran into the lower chamber as described previously. HPMECs were grown on 0.4 *μ*m transwell inserts. Following the indicated time interval for each treatment, 0.5 ml of FITC–dextran (1 mg/ml) was added to the upper wells and 1.5 ml of medium was added to the bottom chamber. After incubation for 1 h in the dark, 50 *μ*l of medium was aspired and measured using a fluorescence plate reader at an excitation wavelength of 488 nm and an emission wavelength of 520 nm. The basal FITC–dextran permeability for unstimulated monolayers was set at 100%.

### EC tube formation assay

EC tube formation assays were conducted for the *in vitro* study of angiogenesis and differentiation according to the manufacturer's instructions. In brief, HPMECs were grown on 48-well plates pre-coated with Matrigel (BD, Franklin Lakes, NJ, USA) at a concentration of 4 × 10^4^ cells per 200 *μ*l. After cell pellets were added to the EGM of corresponding interventions, they were cultured in an incubator at 37 °C with 5% CO_2_ for 12 h and then examined under a phase contrast microscope (BX51 Olympus, Tokyo, Japan) with a 10 × objective. The degree of tube formation was quantified by measuring the lengths of tubes in three randomly chosen fields from each well using ImageJ software (Media Cybernetics, Atlanta, GA, USA).

### EC survival assay

Cell viability was measured using CCK-8. In brief, after cells received their corresponding treatments, cell suspensions were seeded in 96-well plates at 2 × 10^4^ cells per well and pre-incubated at 37 °C in a humidified atmosphere with 5% CO_2_. Then, 10 *μ*l of CCK-8 solution was added to each well of the plate, and the plates were incubated for 2 h in an incubator. The absorbance of each well was measured using a microplate reader at 450 nm (Thermo Scientific, Waltham, MA, USA). Cell viability was calculated using the following equation: viability=(OD_test group_−OD_blank group_)/(OD_control group_−OD_blank group_) × 100%.

### EC apoptosis assay

TUNEL staining and FCM were conducted to evaluate apoptosis. TUNEL was performed using an *in situ* cell death detection kit (Roche Molecular Biology, Mannheim, Germany) according to the manufacturer's instructions. DAPI (KeyGen Biotech) was used for nuclear staining. TUNEL-positive cells were counted in three randomly selected fields of each slide. Following the indicated treatments, the cells were collected and resuspended in 500 ml of 1 × annexin V binding buffer containing 5 *μ*l of annexin V-FITC and 5 *μ*l of propidium iodide (KeyGen Biotech). After the cells were incubated for 10 min at room temperature, they were subjected to FCM (BD) for apoptosis detection.

### Western blot

RIPA buffer (KeyGEN Bio TECH Co., Nanjing, China) was used to extract total protein and Membrane and Cytoplasmic Protein Extraction (KeyGen Biotech) of cells and the left lung tissues of mice in each treatment group following manufacture's instructions.Protein concentrations were measured using a BCA kit. Equivalent amounts of protein (30 *μ*g) were loaded into each well, separated by SDS-PAGE, electro-transferred onto PVDF membranes, blocked with 5% dry milk/BSA and immuno-blotted with the indicated primary antibodies overnight at 4 °C, following by incubation with the correspondent HRP-conjugated secondary antibodies. Using an enhanced chemiluminescence method (Millipore Corporation, Billerica, MA, USA), protein bands were detected using a Bio-Rad Gel Imaging System (Hercules, CA, USA) and analyzed with Quantity One software (Bio-Rad). The expression levels were determined by measuring the corresponding band intensities. The relative values of total protein are expressed normalized to the GAPDH signal. The relative values of memebrane protein are expressed normalized to the pan-cadherin signal.

### IF staining

Coverslips with HPMECs were fixed with 3.7% paraformaldehyde, permeabilized with 0.5% Triton X-100, blocked with PBS containing 5% goat serum and incubated with anti-*β*-catenin, anti-VE-cadherin and anti-NF-*κ*B Rel antibodies at 4 °C overnight, followed by incubation with Alexa Fluor 488-labeled secondary antibodies (Bioworld Technology, Nanjing, China) for 1 h in the dark. Slices were rinsed three times with PBS, and the nuclei were stained with DAPI (KeyGen Biotech) for 5 min. Images were captured using an inverted microscopy (TE2000-U, Nikon, Tokyo, Japan) after the slides were washed.

### Fluorescent phalloidin for F-actin staining

Coverslips with HPMECs were fixed in 3.7% formaldehyde in PBS, rinsed with PBS, permeabilized with 0.1% Triton X-100 in PBS, pre-incubated with PBS containing 1% BSA and stained with 200 *μ*l of fluorescent-iFluor 594 phalloidin solution (KeyGen Biotech) for 30 min at room temperature. After the slides were washed and sealed, the cells were imaged by inverted microscopy (TE2000-U, Nikon).

### Quantitative real-time PCR

Total RNA was isolated from cells using TRIzol reagent (Invitrogen, Carlsbad, CA, USA) according to the manufacturer's instructions and quantified using a Nanodrop 2000 spectrophotometer (Thermo Scientific). Then, 1 *μ*g of total RNA was used as a template for amplifying the cDNA using a HiScript 1st Strand cDNA Synthesis Kit (Vazyme Biotech, Nanjing, China). IL-6 and TNF-*α* gene expression was detected using a HiScript II One Step quantitative real-time PCR SYBR Green Kit (Vazyme Biotech) and a StepOne Real-Time PCR apparatus (Applied Biosystems, Foster City, CA, USA). The relative gene expression was normalized to GAPDH using the comparative Ct (ΔΔCt) method.

### Statistical analysis

Data are present as mean±(S.D.) or for continuous variables that were normally distributed, median (interquartile range) for continuous variables that were abnormally distributed or n for categorical variables. Results are representative of, at least, three independent experiments in triplicate samples.Unpaired Student's *t*-tests s or Mann–Whitney *U* tests were performed, respectively, to compare continuous variables that were normally or abnormally distributed between two independent groups. One-way analysis of variance (ANOVA) followed by the SNK *post hoc* tests or Kruskal–Wallis ANOVA followed by Mann–Whitney *U* tests were performed, resprctively, to compare continuously variables that were abnormally distributed among three or more independent groups and detect significant differences between particular groups. Fisher exact tests were performed for categorical variables. To assess the correlations between omentin levels and the indicated parameters, Spearman rank analysis was used for variables with abnormal distribution and Pearson correlation analysis was used for variables with normal distribution. Statistical significance was set at *P*<0.05 at 95% CI. All statistical analyzes were conducted using GraphPad Prism 5.0 (GraphPad Software, Inc., La Jolla, CA, USA).

## Abbreviations

ARDS: acute respiratory distress syndrome
IJV: internal jugular vein
ELISA: enzyme-linked immunosorbent assay
BALF: bronchoalveolar lavage fluid
LPS: lipopolysaccharide
Ad-omentin: adenoviral vector expressing omentin
Ad-*β*-gal: adenoviral vectors expressing *β*-galactosidase
rh-omentin: recombinant human omentin
ECs: endothelial cells
WBCs: white blood cells
PCT: procalcitonin
H&E: hematoxylin and eosin
EBDA: evans blue-dyed albumin
W/D: wet/dry
HPMEC: human pulmonary microvascular endothelial cell
CCK-8: cell counting kit 8
TUNEL: TdT-mediated dUTP nick end labeling
IF: immunofluorescence
FCM: flow cytometry
AJ: adherens junction
