# Supplementary figures and images for: Omentin protects against LPS-induced ARDS through suppressing pulmonary inflammation and promoting endothelial barrier via an Akt/eNOS-dependent mechanism
Source: Cell Death Dis. 2016 Sep 8;7(9):e2360–. doi: 10.1038/cddis.2016.265 (PMC5059868; doi:10.1038/cddis.2016.265)

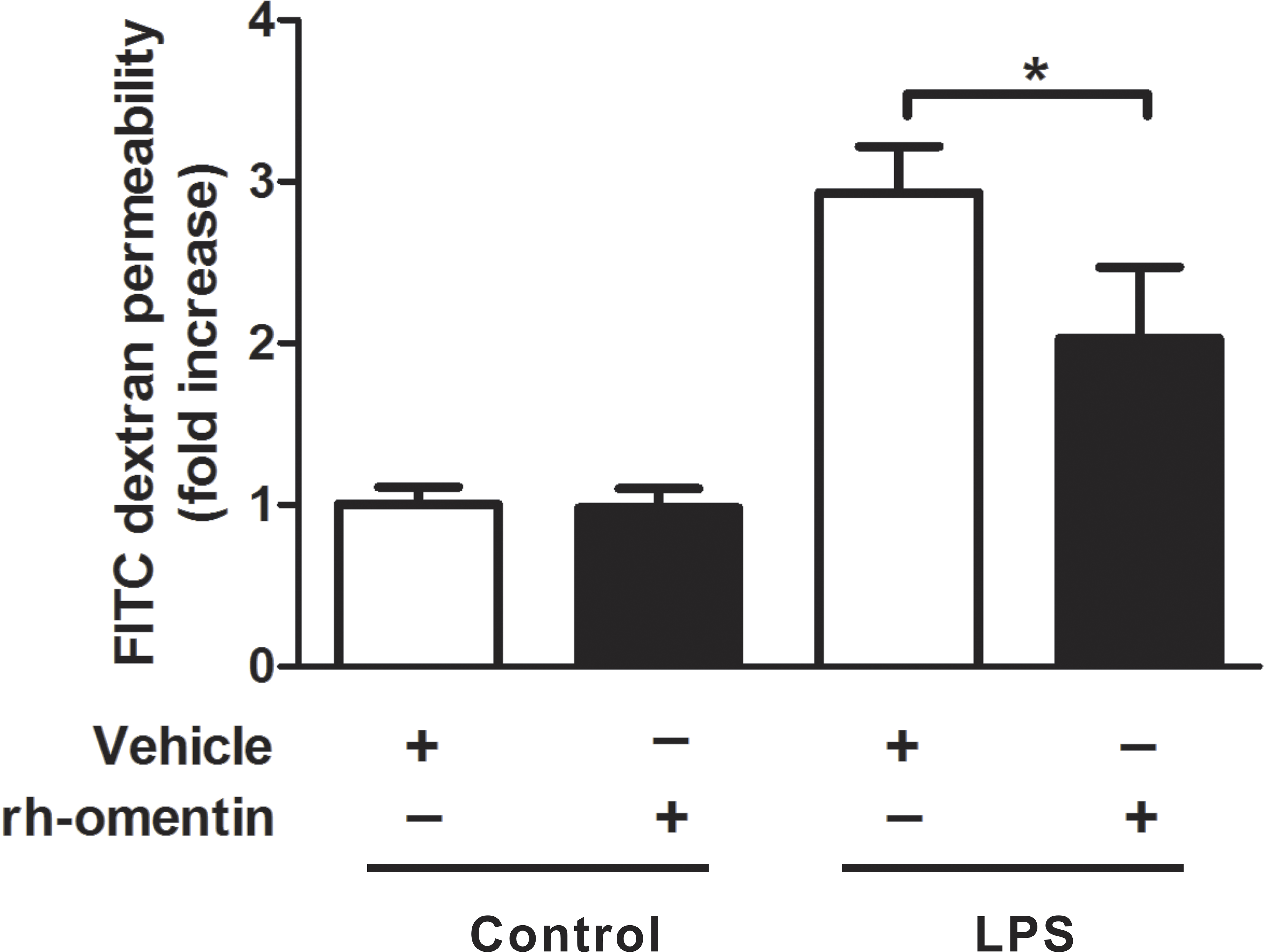

Supplement: Supplementary Figure 1 [file cddis2016265x1.tif]

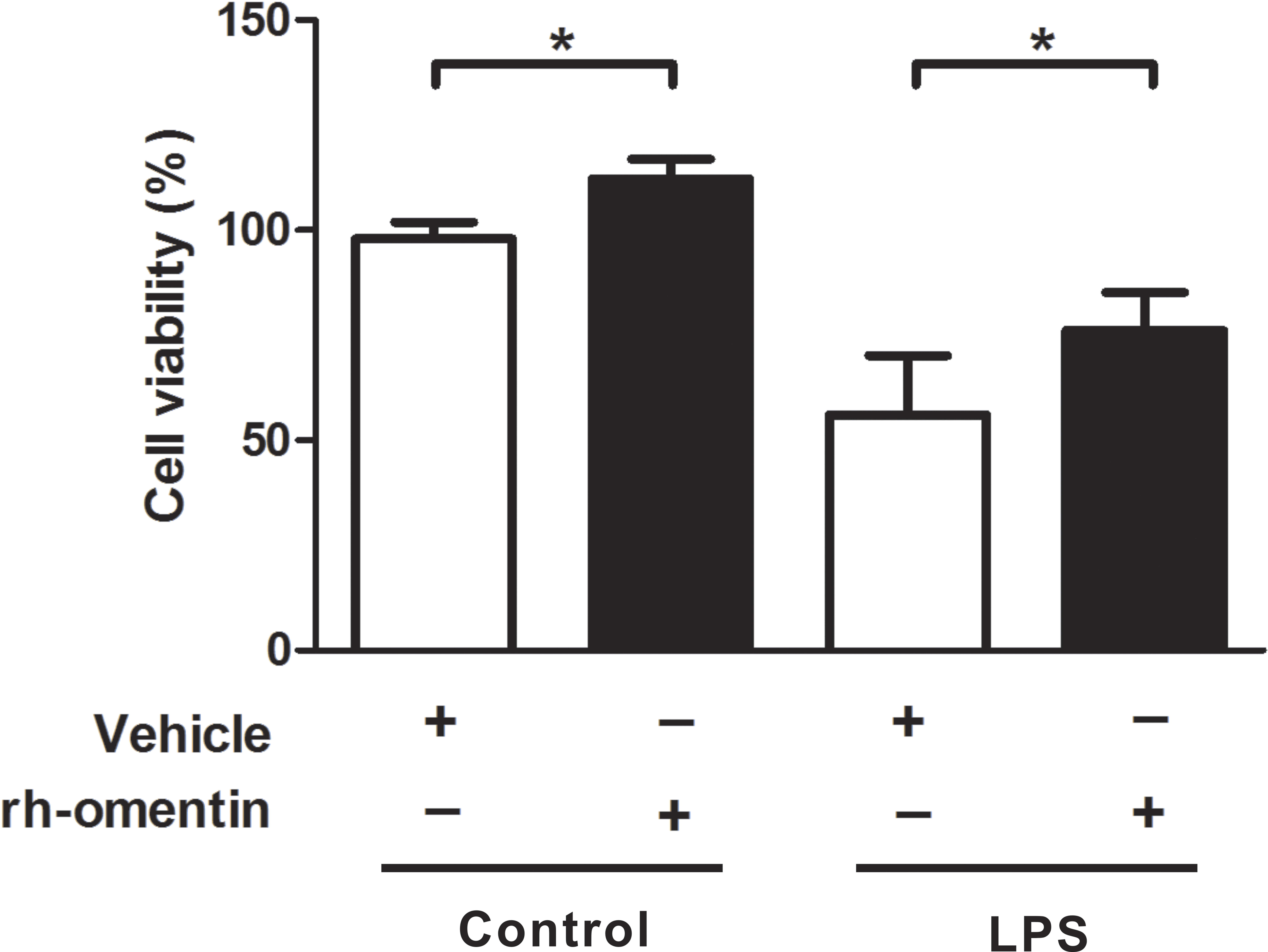

Supplement: Supplementary Figure 2 [file cddis2016265x2.tif]

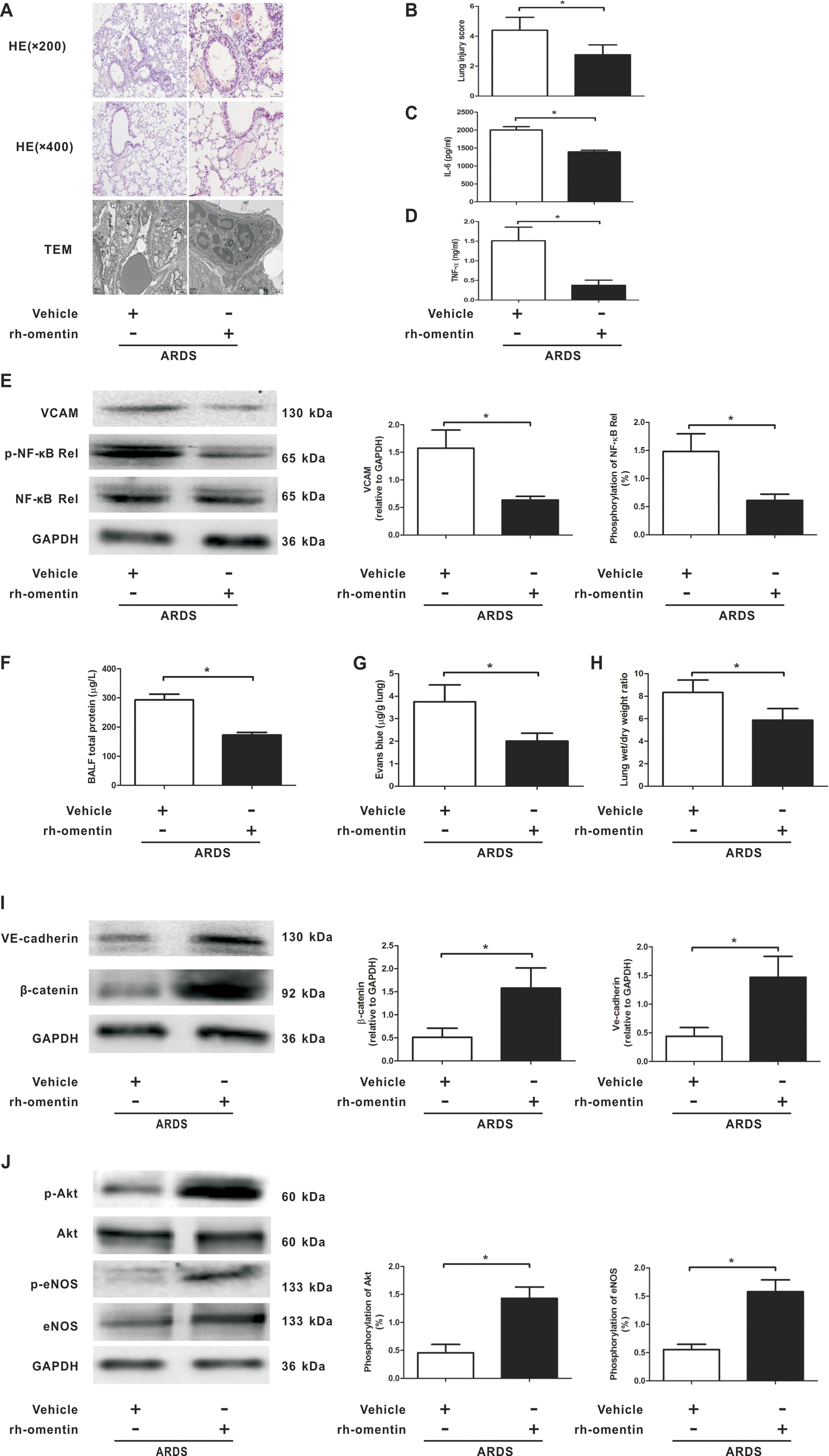

Supplement: Supplementary Figure 3 [file cddis2016265x3.tif]
