# Supplementary material for: Omentin protects against LPS-induced ARDS through suppressing pulmonary inflammation and promoting endothelial barrier via an Akt/eNOS-dependent mechanism
Source: Cell Death Dis. 2016 Sep 8;7(9):e2360–. doi: 10.1038/cddis.2016.265 (PMC5059868; doi:10.1038/cddis.2016.265)
Supplement: Supplementary Figure Legends [file cddis2016265x4.doc]

**Fig. S1 Omentin promotes pulmonary endothelial barrier function after LPS insult *in vitro*.** Administration of rh-omentin prevented the LPS-induced increase in the influx of FITC-dextran. (n=3 independent cultures from each group analyzed in triplicate). HPMECs were cultured with rh-omentin (300 ng/ml) or PBS for 24 h and exposed to either PBS or LPS (100 ng/ml) for 2 h. FITC-dextran (1 mg/ml) was added to the upper wells. After 1 h of incubation in the dark, 50 μl medium from the bottom chamber was aspirated and measured using a fluorescence plate reader. The basal permeability for unstimulated monolayers was set at 100%. The FITC-dextran permeability is expressed as the mean of fold increase ±SD. * p<0.05.

**Figure S2. Omentin improves pulmonary EC survival after LPS insult *in vitro.***

HPMECs were cultured with rh-omentin (300 ng/ml) or PBS for 24 h and then exposed to either PBS or LPS (100 ng/ml) for 2 h. CCK-8 analyses demonstrated that omentin significantly enhanced EC survival after LPS insult *in vitro* (n=3 independent cultures from each group analyzed in triplicate)*.* The data are presented as the mean of fold increase ±SD. * p<0.05.

**Figure S3. One-shot treatment with rh-omentin protein attenuates pulmonary inflammation and endothelial injury after LPS-induced ARDS in mice.**

Mice were subjected to intratracheal injection with LPS (5 mg/kg) or PBS as a control followed by the administration of rh-omentin protein (0.15 μg/g per mouse) at 4 h after LPS insult. **A.** H&E staining showed the histological changes of the lung tissue were alleviated by the administration of rh-omentin in lungs 24 h after LPS instillation (n=5 independent mice from each group assayed in triplicate, magnification, ×200 and ×400). **B.** TEM showed that the ultrastructural pathological damage to pulmonary ECs was mitigated in the omentin-treated group compared with the control group at 24 h after LPS instillation (n=5 independent mice from each group assayed in triplicate). ELISA showed that the administration of rh-omentin decreased the levels of IL-6 (**C**) and TNF-α (**D**) in the lungs at 24 h after LPS instillation (n=6 independent mice from each group assayed in triplicate). **E.** Western blot analysis showed that rh-omentin treatment diminished the levels of VCAM protein and phosphorylated NF-κB Rel in the lungs at 24 h after LPS instillation (n=5 independent mice from each group assayed in triplicate). The BALF protein concentrations (**F**), EBDA extravasation (**G**) and W/D ratio (**H**) were restored by rh-omentin treatment at 24 h after LPS instillation (n=6 independent mice from each group assayed in triplicate). **I.** Western blot analysis showed that rh-omentin treatment elevated the levels of VE-cadherin and β-catenin in the lungs at 24 h after LPS instillation (n=5 independent mice from each group assayed in triplicate). **J.** Western blot showed that rh-omentin treatment increased the phosphorylated Scr levels in the lungs at 24 h after LPS instillation (n=5 independent mice from each group assayed in triplicate). The relative abundances of protein bands were quantified by measuring the corresponding band intensities; the phosphorylation levels of protein are expressed normalized to the total protein signals as shown in the bar graphs. The data are presented as the mean±SD. * p<0.05.
