# Supplementary material for: Omentin protects against LPS-induced ARDS through suppressing pulmonary inflammation and promoting endothelial barrier via an Akt/eNOS-dependent mechanism
Source: Cell Death Dis. 2016 Sep 8;7(9):e2360–. doi: 10.1038/cddis.2016.265 (PMC5059868; doi:10.1038/cddis.2016.265)
Supplement: Supplementary Tables [file cddis2016265x5.doc]

**Table S1**

**Table S1. Clinical Characteristics**

|  | Healthy control (n=35) | ARDS (n=38) |
| --- | --- | --- |
| Age, (years) | 69.31 ± 14.79 | 67.37 ± 14.28 |
| Male/female (n) | 20/15 | 25/13 |
| Body mass index, (kg/m^2^) | 23.82 ± 1.52 | 24.01 ± 1.34 |
| Systolic blood pressure, (mm Hg) | 119.89 ± 5.16 | 118.34 ± 5.42 |
| Fast blood glucose, (mmol/l) | 5.54 ± 0.81 | 5.66 ± 0.63 |
| Total cholesterol, (mmol/l) | 5.16 ± 0.79 | 5.22 ± 0.90 |
| Triglyceride, (mmol/l) | 1.13 ± 0.26 | 1.13 ± 0.20 |
| LDL cholesterol, (mmol/l) | 2.84 ± 0.18 | 2.91 ± 0.25 |
| HDL cholesterol, (mmol/l) | 1.83 ± 0.38 | 1.80 ± 0.24 |
| Omentin, (ng/ml) | 282.46 ± 33.13 | 247.89 ± 83.38^*^ |
| WBC, (10^9^/l) | 7.74 ± 2.35 | 12.96 ± 6.52^*^ |
| Neutrophil counts, (10^9^/l) | 5.55 ± 1.41 | 6.73 ± 1.92 |
| PCT, (ng/ml) | 0.33 ± 0.11 | 4.40 ± 2.59^*^ |
| CRP, (ng/ml) | 7.17 ± 2.24 | 34.07 ± 16.16^*^ |

Values are presented as mean ± SD for continuous variables that are normally distributed or n for categorical variables. * p < 0.05, compared with the healthy controls.

ARDS = acute respiratory distress syndrome; WBC = white blood cell; PCT= procalcitonin; CRT= creative reaction protein; LDL= low-density lipoprotein; HDL= high-density lipoprotein.

**Table S2. Demograhic characteristics and clinical data of 38 patients with ARDS**

|  | Survivor (n=21) | Nonsurvivor (n=17) |
| --- | --- | --- |
| Age, (years) | 66.619 ± 15.42 | 68.29 ± 13.14 |
| Male/female, n | 14/7 | 11/6 |
| APACHE II score, median (IQR) | 22.45 (18.78-27.04) | 26.21 (21.23-29.39)^*^ |
| PaO_2_/FiO_2_ ratio, median (IQR), mm Hg | 94.50 (82.35-132.60) | 82.00 (76.71-89.31)^*^ |
| Etiology of ARDS, n (%) |  |  |
| Pulmonary etiology | 16 (76.19) | 14 (82.35) |
| Pulmonary infection | 13 (61.90) | 11 (64.71) |
| Aspiration | 3 (14.29) | 3 (17.65) |
| Extra-pulmonary etiology | 5 (23.81) | 3 (17.65) |
| Trauma | 2 (9.52) | 1 (5.88) |
| Blood transfusion | 1 (4.76) | 0 (0) |
| Pancreatitis | 2 (9.52) | 1 (5.88) |
| Comorbidity, n (%) |  |  |
| Obstructive airway disease | 7 (33.33) | 8 (47.06) |
| Hypertension | 9 (42.86) | 9 (52.94) |
| Diabetes | 4 (19.05) | 6 (35.29) |
| Cardiovascular disease | 4 (19.05) | 3 (17.65) |
| Cerebrovascular accident | 1 (4.76) | 2 (11.76) |
| Cancer history | 4 (19.05) | 2 (11.76) |
| Hematological disease | 0 (0) | 1 (5.88) |
| Chronic renal failure | 0 (0) | 1 (5.88) |
| ARDS severity, n (%) |  |  |
| Mild (n=11) | 7 (33.33) | 4 (23.53) |
| Morderate (n=19) | 11 (52.38) | 8 (47.05) |
| Severe (n=8) | 3 (14.29) | 5 (29.41) |
| Body mass index, mean ± SD, kg/m^2^ | 23.71 ± 1.30 | 24.39 ± 1.33 |
| Systolic blood pressure, mean ± SD, mm Hg | 119.14 ± 5.71 | 117.35± 5.01 |
| Fast blood glucose, mean ± SD, mmol/l | 5.66 ± 0.63 | 5.67 ± 0.66 |
| Total cholesterol, mean ± SD, mmol/l | 5.33 ± 0.93 | 5.08 ± 0.87 |
| Triglyceride, mean ± SD, mmol/l | 1.16 ± 0.19 | 1.08 ±0.20 |
| LDL cholesterol, mean ± SD, mmol/l | 2.94 ± 0.24 | 2.89 ± 0.27 |
| HDL cholesterol, mean ± SD, mmol/l | 1.84 ± 0.22 | 1.73 ± 0.25 |
| Omentin, mean ± SD, ng/ml | 297.46 ± 79.18 | 186.66 ±32.10^*^ |
| White blood cell, mean ± SD, 10^9^/l | 10.40 ± 5.72 | 16.12 ± 6.20^*^ |
| Neutrophil counts, mean ± SD, 10^9^/l | 6.86 ± 2.24 | 6.56 ± 1.48 |
| PCT, mean ± SD, ng/ml | 3.42 ± 1.96 | 5.60 ± 2.81^*^ |
| CRP, mean ± SD, ng/ml | 28.41 ± 14.44 | 41.05 ± 15.79^*^ |
| Duration of mechanical ventilation, median (IQR), day | 12.00 (9.75-16.25) | 15.00 (12.00-19.50)^*^ |
| Length of ICU stay, median (IQR), day | 15.00 (12.00-18.00) | 14.00 (12.00-18.50) |
| Length of hospital stay, median (IQR), day | 19.50 (14.75-25.25) | 15.00 (13.00-20.00)^*^ |
| Organ dysfunction, n (%) |  |  |
| Septic shock | 4 (19.05) | 9 (52.94) |
| Renal failure | 3 (14.29) | 8 (47.06) |
| Coagulopathy | 0 (0) | 6 (35.29) |
| Hepatic failure | 5 (23.81) | 6 (35.29) |

Values are presented as mean ± SD for continuous variables that are normally distributed, median (IQR) for continuous variables that are not normally distributed or n for categorical variables. * p < 0.05, compared with the survivors.

ARDS = acute respiratory distress syndrome; APACHE = acute physiology and chronic health evaluation; PaO_2_/FiO_2_ ratio = the ratio of partial pressure of arterial oxygen (PaO_2_) to the fraction of inspired oxygen (FiO_2_); LDL= low-density lipoprotein; HDL= high-density lipoprotein; WBC = white blood cell; PCT= procalcitonin; CRP = C-reactive protein
